# Development and external validation of a nomogram to predict the risk of Upper gastrointestinal precancerous lesions in a non‐high‐incidence area

**DOI:** 10.1002/cam4.3462

**Published:** 2020-09-16

**Authors:** Hai‐Fan Xiao, Shi‐Peng Yan, Ji‐Gang Li, Zhao‐Hui Shi, Yan‐Hua Zou, Ke‐Kui Xu, Xian‐Zhen Liao

**Affiliations:** ^1^ The Department of Cancer Prevention and Control Hunan Cancer Hospital and the Affiliated Cancer Hospital of Xiangya School of Medicine Central South University Changsha China; ^2^ Department of Pathology Hunan Cancer Hospital and the Affiliated Cancer Hospital of Xiangya School of Medicine Central South University Changsha China

**Keywords:** endoscopy screening, external validation, nomogram, non‐high‐incidence area, upper gastrointestinal precancerous lesions

## Abstract

**Background:**

Upper gastrointestinal precancerous lesions (UGPL) is the major preventable disease in non‐high‐incidence area. A prognostic nomogram was constructed to predict and identity susceptible population of UGPL before endoscope screening.

**Methods:**

We recruited 300 ,016 eligible participants for upper gastrointestinal cancer (UGC) screening aged 40‐74 years from two cities in Hunan province from 2012 to 2019. Individuals at high risk of UGC on basis of questionnaire estimation underwent endoscopic screening. Participants in two cities accepting endoscopy were used as training and external validation cohorts, respectively. A nomogram was developed based on independent prognostic factors of UGPL determined in multivariable logistic regression analysis.

**Results:**

Of 35, 621 with high risk for UGC, 10, 364 subjects undertook endoscopy (participation rate of 29.1%). The detection rate for UGPL was 4.55%. The nomogram showed that age, gender, mental trama, picked food, and atrophic gastritis history in a descending order were significant contributors to UGPL risk. The C‐index value of internal and external validation of the model is 0.612 and 0.670, respectively. The calibration data for UGPL showed optimal agreement between the nomogram prediction and actual observation. Furthermore, high‐risk and low‐risk group divided based on score from the nomogram predicted a significantly distinct detection rate.

**Conclusion:**

The nomogram provides screening workers a simple and accurate tool for identifying individuals at a higher risk of UGPL as primary screening before endoscopy among Chinese population in non‐high‐risk areas, thus reducing the incidence of UGC by improving the UGPL detection.

## Graphical abstract

We performed a cross‐sectional study from October 2012 with the major national public health project—Early Screening Program in Urban China (CanSPUC) in Hunan province. In this study, we analyze the results of upper gastrointestinal cancer (UGC) screening conducted in the first 7 year of CanSPUC. And for the firstly time, we aim to develop and externally validate a nomogram for predicting the risk of UGPL, so as to provide a simple and accurate tool for risk questionnaire evaluation of UGC screening in non‐high‐incidence area.

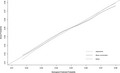

## 1 BACKGROUND

Upper gastrointestinal cancer (UGC) containing esophageal cancer (EC) and gastric cancer (GC) is one of the most common cancers with more than 1.6 million new cases and 1.3 million deaths in 2018 worldwide.^1^ China is a high‐risk country for UGC accounting for about 50% (0.76 million new cases, 0.67 million deaths in 2018) of the global burden. Moreover, UGC is the second cancer both in the incidence and death spectrum (only next to lung cancer) in China.^2^ Meanwhile, most UGC cases present in advanced stages and the overall 5‐year survival is below 36%.^3^ But if the patients were diagnosed at early stage of UGC, the 5‐year survival rate would be substantially improved.^4^ Therefore, early detection and treatment have great potential to improve survival and reduce disease mortality.^5^

Upper endoscopy with biopsy, the gold‐standard for diagnosis of UGC, has been used in many countries such as Japan, Korea, and China as a screening method.^6^, ^7^, ^8^ Many studies showed that endoscopic screening might be associated with reduced mortality on UGC in some areas of Asia with high incidence.^8^, ^9^, ^10^, ^11^, ^12^ Therefore, China initiated a major public health program named Cancer Screening Program in Urban China (CanSPUC) in 2012 which was implemented in nationwide including high‐incidence and non‐high‐incidence areas.^13^ UGC screening in the CanSPUC was carried out through two‐step screening method—a questionnaire combined with endoscopy. Within China, striking geographic variations exist in the incidences of UGC, which can vary more than 10‐fold in different regions.^14^, ^15^ Hunan province, as non‐high‐incidence area, also stated this program at 2012 with serious disease burden of UGC.^16^ Our previous cluster randomized trial showed that the questionnaire evaluation could not effectively concentrate the UGC high‐risk individuals.^16^ Furthermore, risk factors of this questionnaire evaluation are similar to the CanSPUC whose questionnaire was designed mostly based on the evidence from high‐incidence area in China. So the questionnaire evaluation is probably not suitable for the people in non‐high‐incidence area, and we need a more simple and accurate evaluation system as primary screening tool.

Using a graphical calculator, nomograms have been used to build predictive models that serve as popular mathematical tools based on regression models. Nomograms are accurate and precise tools by creating an intuitive graph of a statistical predictive model for estimating risk, by correlating the relationship between parameters and various cancer prognosis parameters such as metastatic probability, overall survival (OS), and disease risk.^17^, ^18^ In our previous study, precancerous lesions (PL) of UGC screening are the major the detection result in non‐high‐incidence area. And to data, there has never been a study intended to develop a nomogram to predict the risk of UGPL. Therefore, in this study, we analyze the results of UGC screening conducted in the first 7 year of CanSPUC in Hunan province in China from October 2012 to October 2019. And for the first time, we aim to develop and externally validate a nomogram for predicting the risk of UGPL, so as to provide a simple and accurate tool for risk questionnaire evaluation of UGC screening in non‐high‐incidence area.

## 2 MATERIALS AND METHODS

### 2.1 Study design and population

We performed a cross‐sectional study from October 2012 with the major national public health project—Early Screening Program in Urban China (CanSPUC). For the present analysis, we used the data of 7 years with UGC screening until October 2019, which covered two cities (Changsha and Yueyang city) in Hunan province. Ethical approval was acquired from the Ethics Committee of Cancer Hospital Chinese Academy of Medical Sciences. And it was performed in accordance with the Declaration of Helsinki.

Our study was conducted in communities of cities. Most of individuals signed up the project as community advertisement and media publicity. Also, general practitioners (GPs) contacted every individual by telephone or a home visit to invite them to participate on basis of age‐eligible individual roster in each community. Every subject need to sign informed consent. The inclusion criteria were: (a) subjects are local residents aged 40‐74 years old; (b) with no previous history of cancer; and (c) generally in good condition mentally and physically and can sign the informed consent form.

### 2.2 Screening procedures

#### 2.2.1 Risk assessment

All eligible participants first need to fill a unified questionnaire as preliminary screening. Only those who were assessed to be at high risk of UGC were then advised to take endoscopy screening free of charge. The high risk result presented immediately after input the questionnaire survey according to electronically analysis of the cancer risk score system. Based on the Harvard Risk Index this system was developed through adjusting some involving parameters (risk factors, relative risks, and exposure rates of risk factors) according to Chinese people's characteristics.^19^ The risk scoring system covered risk factors as follows: body mass index (BMI), drinking history, high‐salt diet intake, more‐dry diet intake, mental trauma history, history of chronic gastritis, history of duodenal ulcer, smoking history, drinking tea history, pickled food intake, more‐hot diet intake, indoor soot exposure in the past 10 years, history of upper gastrointestinal system diseases and family history of esophageal or gastric cancer. After the discussion of multidisciplinary expert arm based on epidemiological data in China, each risk factor was assigned a score based on the magnitude of its association with esophageal or gastric cancer.

#### 2.2.2 Endoscopic screening

All high‐risk subjects were suggested to take endoscopic screening at the two designed tertiary‐level hospitals. Before endoscopy routine coagulation function and electrocardiogram only for people over 60 years old were conducted. Endoscopy was performed in strict accordance with “Technical program of UGC screening and early diagnosis and treatment project (2014 trial version)”.^20^ During the endoscopy screening, if any positive or suspicious lesion is detected, biopsy needs to be taken. The detailed requirements see our previous study.^16^ We freely provided to subjects for normal endoscopy performed under without anesthesia.

#### 2.2.3 Outcome ascertainment and Re‐examination

Upon pathologically diagnosing, cancer contains carcinoma in situ, EC, GC, and other cancers; Precancerous lesions includes severe dysplasia (high‐grade intraepithelial neoplasia), mild esophageal dysplasia requiring re‐examination once every 3 years, moderate esophageal dyspepsia, cardiac or gastric low‐grade intraepithelial neoplasia and severe intestinal metaplasia (those three kind of PL requires re‐examination annually). So some special subjects will have more than once endoscopy as re‐examination. In the present study, UGPL includes PL and caner.

#### 2.2.4 Quality control

GPs and relative physician of clinical screening department in hospital were responsible for collecting paper‐based standardized documentation forms including risk assessment questionnaire, endoscopy screening forms and pathology diagnosis forms. Then, these data were entered electronically into the information system. There levels of quality control were performed to ensure the data accuracy: 1) after the first input of data, a quality controller who was designed in every community or clinical screening department make the first accuracy check; 2) then the information system identity missing or incorrect contents with electronically logical self‐inspection function; 3) the data management center in National Cancer Center of China where all data were submitted to perform quality control periodically.

### 2.3 Statistical analysis

In present study, the nomogram was designed to give the risk probability on precancerous lesions or above of UGC. We used the Changsha city data as training dataset for constructing nomogram model, and Yueyang city data as external validation dataset. Categorical variables were analyzed using χ^2^ test (or Fisher's exact test). To reduce the dimension of data, we used the least absolute shrinkage and selection operator (LASSO) method to select optimal features. Through LASSO regression, the prediction model incorporating factors selected was constructed on basis of multivariate logistic regression analysis. All factors with *P* value < .05 were included in the prediction model.

Using 1000 bootstrap resamples internal validation of the training dataset was performed and external validation was performed taking the validation cohort on the nomogram. The concordance index (C‐index) was the index of evaluating the performance of the model to prognostically predict outcomes. The nomogram was calibrated by comparing the predicted rates of precancerous lesions or above of UGC with the observed rates. The sum‐score for each subject was calculated based on the established model. Subjects were then divided as high‐risk and low‐risk group. Risk score above 127 was considered as high risk for UGC. The optimal cutoff value was determined by a receiver operating characteristic (ROC) curve.

All data management and statistical analyses were performed using the R software, version 3.6.3 (R Foundation for Statistical Computing, Vienna, Austria). All statistical tests were two‐sided, and a *P*‐value < .05 was considered statistically significant.

## 3 RESULTS

### 3.1 Characteristics of the study population

**Figure 1:**
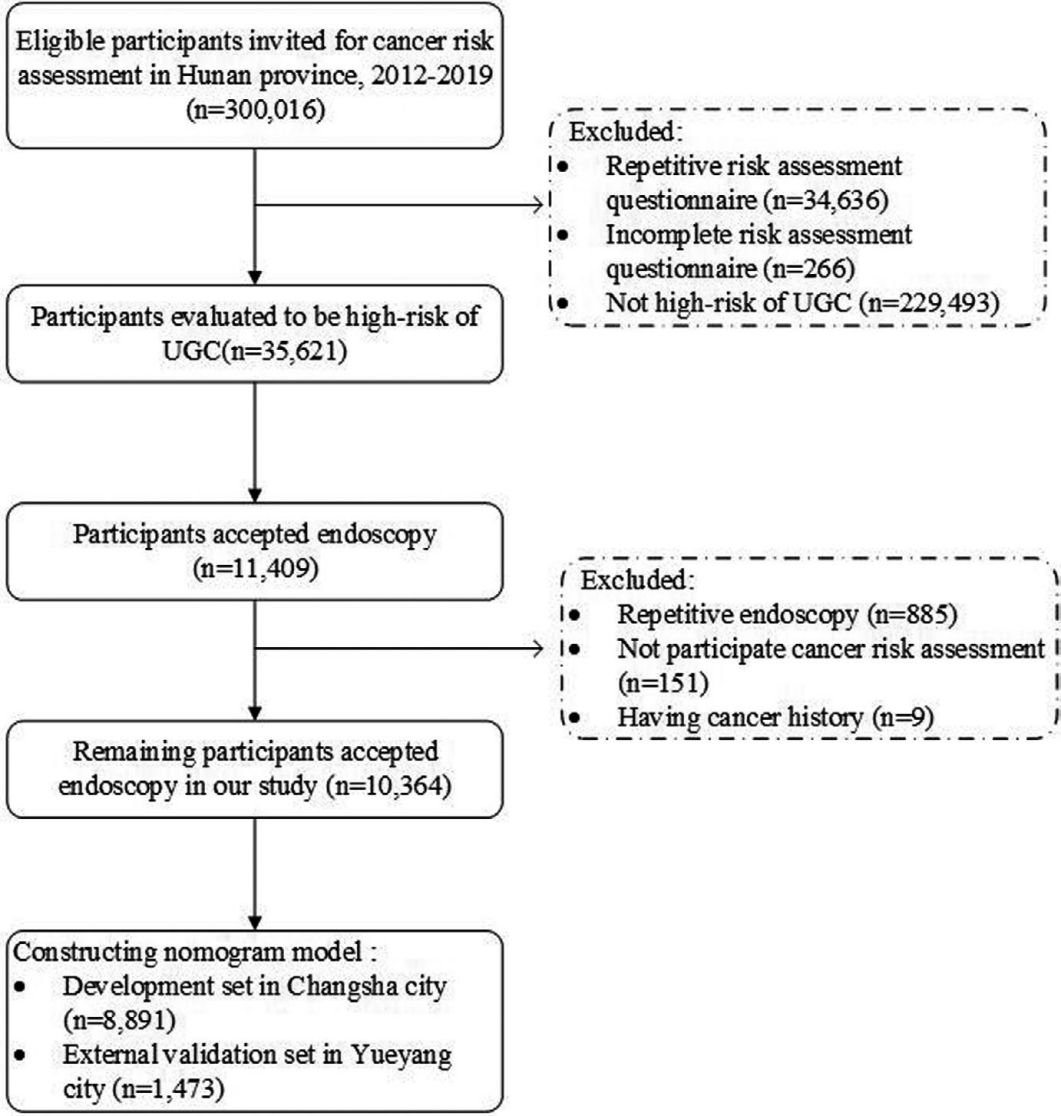
Flow diagram of participant recruitment

There were 300,016 eligible participants recruited in the CanSPUC from 2012 to 2019. After data cleaning, we excluded participants with repetitive risk assessment questionnaire (n = 34,636), incomplete risk assessment questionnaire (n = 266) and risk assessment results showing not high risk for UGC (n = 229,493). Then, 35,621 remaining participants of high risk for UGC were included, with high‐risk rate of 11.9%. Among them, a total of 11,409 individuals accepted endoscopy screening for UGC. Those who had repetitive endoscopy as re‐examination (n = 885), not participated cancer risk assessment as some violated the screening process (n = 151) and had cancer history (n = 9) were excluded in our final analysis. Finally, remaining 10,364 accepted endoscopy in our study, with participation rate of 29.1%. Overall, 472 UGPL cases were diagnosed (4.55%). Among them, 372 cases (3.79%) were pathologically diagnosed as GCPL or above (304 of low‐grade intraepithelial neoplasia, 44 of indeterminate intraepithelial neoplasia, 3 of high‐grade intraepithelial neoplasia, 3 of glandular epithelial intramucosal adenocarcinoma, 2 of invasive adenocarcinoma and 16 of severe intestinal metaplasia), and 101 cases (1.03%) were diagnosed as ECPL or above (83 of mild esophageal dysplasia, 7 of moderate esophageal dyspepsia, 2 of severe dysplasia, 3 of dysplasia cannot be classified, 3 of carcinoma in situ, 1 of intramucosal squamous cell carcinoma and 2 of invasive squamous cell carcinoma). Among 10,364 participants undertaking endoscopy, 8,891 individuals in Changsha city was designed as development set for constructing nomogram and 1,473 in Yueyang city was taken as external validation set. The detailed flow of the study is shown in Figure 1.

The characteristics and potential risk factors for UGPL of endoscopy screening participates are presented in Table 1. In the UGPL training set, there are 3717 men (41.8%) and 5174 women (58.2%). The mean age was 56.13 years (SD = 8.09 years), and the majority (20.5%) were between 60 and 64 years old. About 29.5% had history of reflux esophagitis, 77.1% had history of superficial gastritis, 35.9% had atrophic gastritis previously, and 33.1% had history of gastric or duodenal ulcers. As shown in Table 1, we found the detection rate of UGPL were statistically significant between the groups of age, BMI, gender, picked food intake, mind trauma recently and history of atrophic gastritis (*P* < .05). We also analyzed the detection rate of GCPL and ECPL respectively between the groups of demographic characteristic variables and potential risk factors (See the Table S1).

**Table 1:** Demographic characteristic variables and potential risk factors for upper gastrointestinal precancerous lesions

Variables		UGPL training set (n = 8891)			UGPL external validation set (n = 1473)		
		Case, %	Negative, %	*P*	Case, %	Negative,%	*P*
All cases		428 (4.8)	8463 (95.2)		44 (3.0)	1429 (92.0)	
Age^†^	$\bar{x}$ ± s	57.9 ± 7.7	55.8 ± 8.1	<.001	57.0 ± 7.8	53.3 ± 8.2	.003
Age group (Year)	40‐44	30 (3.6)	804 (96.4)	<.001	4 (1.6)	251 (98.4)	.090
	45‐49	37 (3.0)	1223 (97.0)		4 (1.3)	301 (98.7)	
	50‐54	74 (4.2)	1685 (95.8)		9 (3.1)	281 (96.9)	
	55‐59	76 (4.7)	1534 (95.3)		9 (4.4)	196 (95.6)	
	60‐64	107 (5.9)	1712 (94.1)		9 (3.7)	237 (96.3)	
	65‐74	104 (6.5)	1505 (93.5)		9 (3.3)	163 (96.7)	
Body mass index (Kg/m^2^)	<18.5	21 (7.7)	252 (92.3)	.021	0	47 (100.0)	.836
	18.5‐23.9	235 (4.4)	5152 (95.6)		29 (3.2)	876 (96.8)	
	24‐26.9	127 (5.2)	2333 (94.8)		11 (3.0)	361 (97.0)	
	≥27	45 (5.8)	726 (94.2)		4 (2.7)	145 (97.3)	
Gender	Male	225 (6.1)	3492 (93.9)	<.001	27 (4.3)	595 (95.7)	.014
	Female	203 (3.9)	4971 (96.1)		17 (2.0)	834 (98.0)	
Marital status	Married	15 (4.2)	340 (95.8)	.688	42 (3.0)	1383 (97.0)	.652
	Unmarried	413 (4.8)	8123 (95.2)		2 (4.2)	46 (95.8)	
Education	Primary school or below	63 (4.4)	1361 (95.6)	.747	7 (2.4)	286 (97.6)	.621
	Middle school	291 (4.9)	5685 (95.1)		31 (3.3)	904 (96.7)	
	College degree or above	74 (5.0)	1417 (95.0)		6 (2.5)	239 (97.5)	
Occupational exposure	Yes	308 (5.0)	5841 (95.0)	.218	39 (2.9)	1297 (97.1)	.596
	No	120 (4.4)	2622 (95.6)		5 (3.7)	132 (96.3)	
Smoking	Current	160 (5.5)	2751 (94.5)	.106	15 (5.9)	239 (94.1)	.227
	Never	249 (4.5)	5335 (95.5)		29 (2.4)	1190 (97.6)	
	Former	19 (4.8)	377 (95.2)		0	0	
Drinking	Current	169 (4.9)	3256 (95.1)	.672	9 (4.6)	186 (95.4)	.005
	Never	242 (4.7)	4925 (95.3)		35 (2.7)	1243 (97.3)	
	Former	17 (5.7)	282 (94.3)		0		
Vegetable intake	Frequently	289 (4.6)	6014 (95.4)	.129	41 (2.9)	1393 (97.1)	.108
	Rarely	139 (5.4)	2449 (94.6)		3 (7.7)	36 (92.3)	
Fruit intake	Frequently	89 (4.9)	1744 (95.1)	.974	31 (3.1)	1010 (96.9)	.913
	Rarely	339 (4.8)	6719 (95.2)		13 (2.8)	450 (97.2)	
Meat intake	Frequently	191 (5.2)	3488 (94.8)	.178	39 (3.1)	1207 (96.7)	.587
	Rarely	237 (4.6)	4975 (95.4)		5 (2.2)	222 (97.8)	
Coarse Food Grain intake	Frequently	84 (5.0)	1600 (95.0)	.758	13 (3.1)	411 (96.9)	.999
	Rarely	344 (4.8)	6863 (95.2)		31 (3.0)	1018 (97.0)	
Pickled food intake	Frequently	144 (5.7)	2374 (94.3)	.014	15 (4.8)	297 (95.2)	.052
	Rarely	284 (4.5)	6089 (95.5)		29 (2.5)	1132 (97.5)	
High‐temp food intake	Frequently	158 (4.7)	3224 (95.3)	.660	5 (2.4)	207 (97.6)	.717
	Rarely	270 (4.9)	5239 (95.1)		39 (3.1)	1222 (96.9)	
Salty food intake	Frequently	228 (4.9)	4398 (95.1)	.633	21 (3.3)	607 (96.7)	.590
	Rarely	200 (4.7)	4065 (95.3)		23 (2.7)	822 (97.3)	
Dry food intake	Frequently	151 (4.6)	3112 (95.4)	.567	2 (1.8)	111 (98.2)	.574
	Rarely	277 (4.9)	5351 (95.1)		42 (3.1)	1318 (96.9)	
Fried food intake	Frequently	202 (4.7)	4097 (95.3)	.659	2 (1.6)	125 (98.4)	.581
	Rarely	226 (4.9)	4366 (95.1)		42 (3.1)	1304 (96.9)	
Mental trauma recently	Yes	108 (3.9)	2648 (96.1)	.010	1 (1.6)	61 (98.4)	.999
	No	320 (5.2)	5815 (94.5)		43 (3.1)	1368 (96.9)	
Chronic mental depression	Yes	118 (4.2)	2701 (95.8)	.067	3 (4.7)	61 (95.3)	.436
	No	310 (5.1)	5762 (94.9)		41 (2.9)	1368 (97.1)	
Reflux esophagitis	Yes	133 (5.1)	2494 (94.9)	.512	3 (6.5)	43 (93.5)	.155
	No	295 (4.7)	5969 (95.3)		41 (2.9)	1386 (97.1)	
Superficial gastritis	Yes	325 (4.7)	6533 (95.3)	.585	11 (2.7)	400 (97.3)	.791
	No	103 (5.1)	1930 (94.9)		33 (3.1)	1029 (96.9)	
Atrophic gastritis	Yes	178 (5.6)	3018 (94.4)	.015	1 (4.8)	20 (95.2)	.473
	No	250 (4.4)	5445 (95.6)		43 (3.0)	1409 (97.0)	
Gastric and duodenal ulcers	Yes	290 (4.9)	5665 (95.1)	.765	9 (3.8)	227 (96.2)	.545
	No	138 (4.7)	2798 (95.3)		35 (2.8)	1232 (97.2)	
Family history of upper gastrointestinal cancer	Yes	296 (4.8)	5906 (95.2)	.824	2 (2.9)	68 (97.1)	.999
	No	132 (4.9)	2557 (95.1)		42 (3.00)	1361 (97.0)	

^†^ Variables were described by mean (**x**) and standard deviation(**s**).

### 3.2 Feature selection

Based on LASSO method, we selected factors to enter multivariate logistic analysis. Of the demographic and potential risk factors, eight potential predictors that had non‐zero coefficients were identified and selected from 24 features of UGPL (Figure S1A,B). The selected features were age group, gender, occupational exposure, meat intake frequently, vegetable intake frequently, mental trauma recently and atrophic gastritis history. For GCPL, we also selected eight potential predictors from 24 features (Figure S1C,D). They are age group, gender, occupational exposure, picked food intake frequently, vegetable intake frequently, chronic mental depression, mental trauma recently, and atrophic gastritis history. For ECPL, six potential predictors were chose from 23 features (Figure S1E,F)—age group, gender, salty food intake frequently, fruit intake frequently, and EC family history.

### 3.3 Multivariate analysis and establishment of prediction model

Parameters selected through LASSO regression were entering into the multivariate logistic analysis. We found age group, Male gender, picked food intake frequently, atrophic gastritis history, and mental trauma history were independently associated with the risk of UGPL. Age group, male gender, picked food intake frequently, and vegetable intake frequently were significantly related with a higher risk of GCPL. From the variable of age group, the results indicated the risk of UGPL and GCPL increased with the older age. And male gender were salty food intake frequently found to be correlated with the risk of ECPL (see the Table 2).

**Table 2:** Multivariate logistic analysis of risk factors for upper gastrointestinal, esophageal, and gastric precancerous lesions

Variables		UGPL and above		GC and above		EC and above	
		Adjusted OR (95% CI)	*P*	Adjusted OR (95% CI)	*P*	Adjusted OR (95% CI)	*P*
Age group^†^	40‐44 /40‐49	Ref		Ref		—	
	45‐49 /50‐59	0.82 (0.33‐1.31)	.419	1.39 (0.78‐1.99)	.289	—	
	50‐54 /60‐74	1.21 (0.77‐1.64)	.396	1.85 (1.29‐2.41)	.031	—	
	55‐59	1.37 (0.94‐1.81)	.150	2.08 (1.52‐2.64)	.010	—	
	60‐64	1.67 (1.25‐2.08)	.007	2.54 (2.00‐3.08)	.001	—	
	65‐74	1.78 (1.36‐2.20)	.021	2.53 (1.98‐3.07)	.001	—	
Gender	Female	Ref		Ref		Ref	
	Male	1.52 (1.32‐1.71)	.000	1.46 (1.24‐1.68)	.001	1.87 (1.46‐2.29)	.003
Pickled food	Yes	1.30 (1.09‐1.51)	.015	1.41 (1.18‐1.65)	.004	—	
	No	Ref		Ref		—	
Atrophic gastritis	Yes	1.27 (1.07‐1.47)	.020	—		—	
	No	Ref		—		—	
Mental trauma recently	Yes	0.75 (0.53‐0.98)	.013	—		—	
	No	Ref		—		—	
Vegetable intake	Yes	—	—	0.77 (0.56‐0.99)	.036	—	
	No	—	—	Ref		—	
Salty food intake	Yes	—	—	—	—	2.0 (1.53‐2.41)	.003
	No	—	—	—	—	Ref	

Ref was reference subgroup.

^†^ Age group in EC and above analysis were divided in three groups as few positive cases.

**Figure 2:**
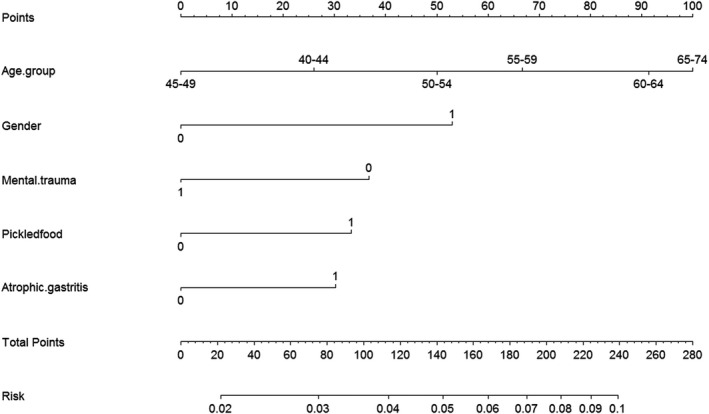
*Prognostic nomogram for the risk of upper gastrointestinal precancerous lesions*

For the high‐risk assessment of GC or EC, we used the same and unified assessment questionnaire of UGC, so one nomogram for prediction the risk of UGPL was established using those independently factors. The nomogram plot showed that age group was the greatest contributing factor to the risk of UGPL. Other factors that significantly contributed to UGPL risk to a lesser degree were male gender, mental trauma history, picked food intake frequently, and atrophic gastritis history (Figure 2).

### 3.4 Calibration and validation of the nomogram

**Figure 3:**
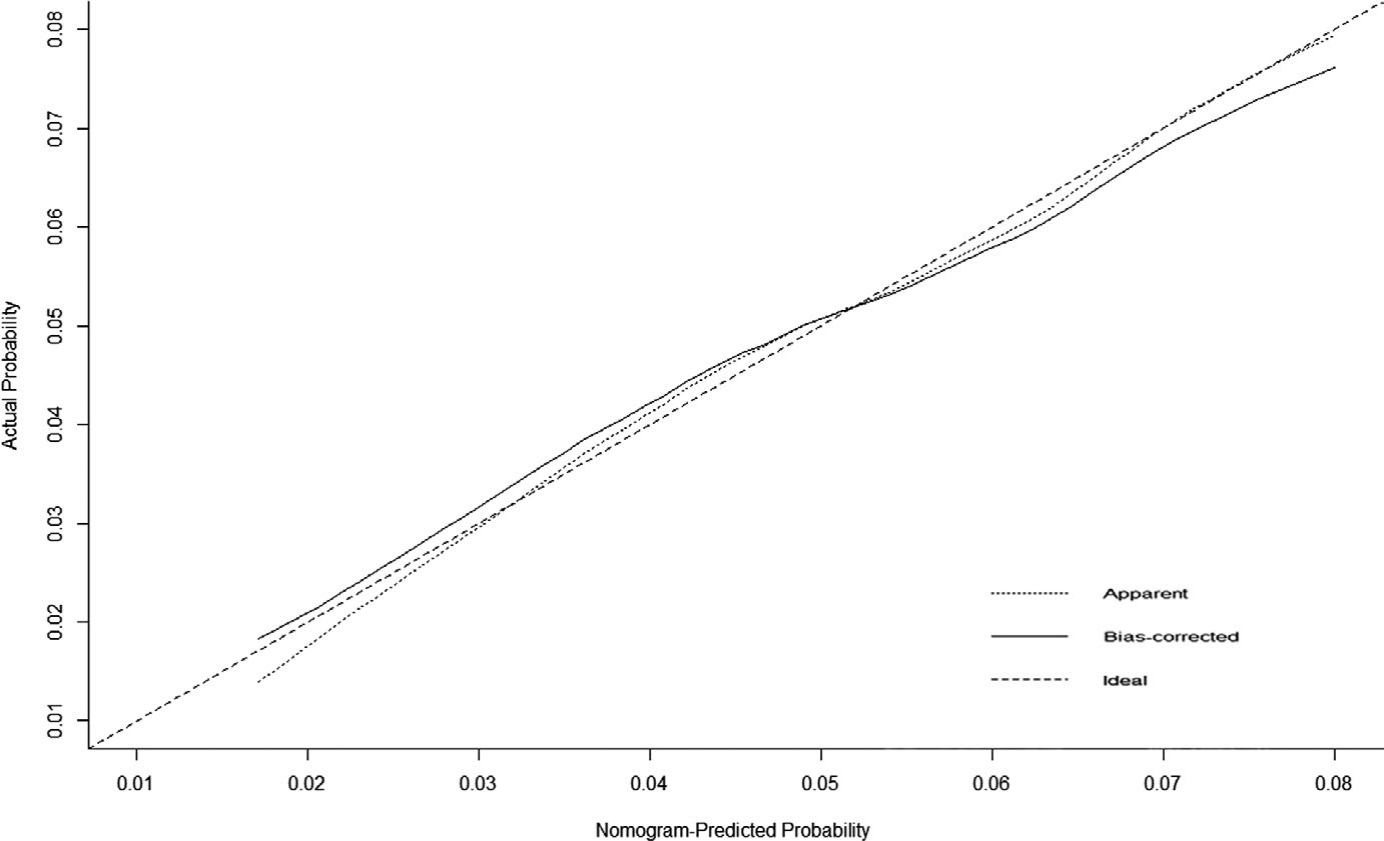
The calibration curves for predicting upper gastrointestinal precancerous lesions

The calibration data for UGPL plots had optimal agreement between the nomogram prediction and actual observation, with the range from 0.01 to 0.08 (Figure 3). Internal validation of the nomogram model yielded a C‐index value of 0.612 (0.585‐0.639) for UGPL. Data from Yueyang city (n = 1473) were used for external validation of the nomogram model, which yielded a C‐index value of 0.670 (0.587‐0.753) for UGPL, which was higher than the index of the development group (*P* < .05).

### 3.5 Performance of the nomogram in stratifying participant risk

**Figure 4:**
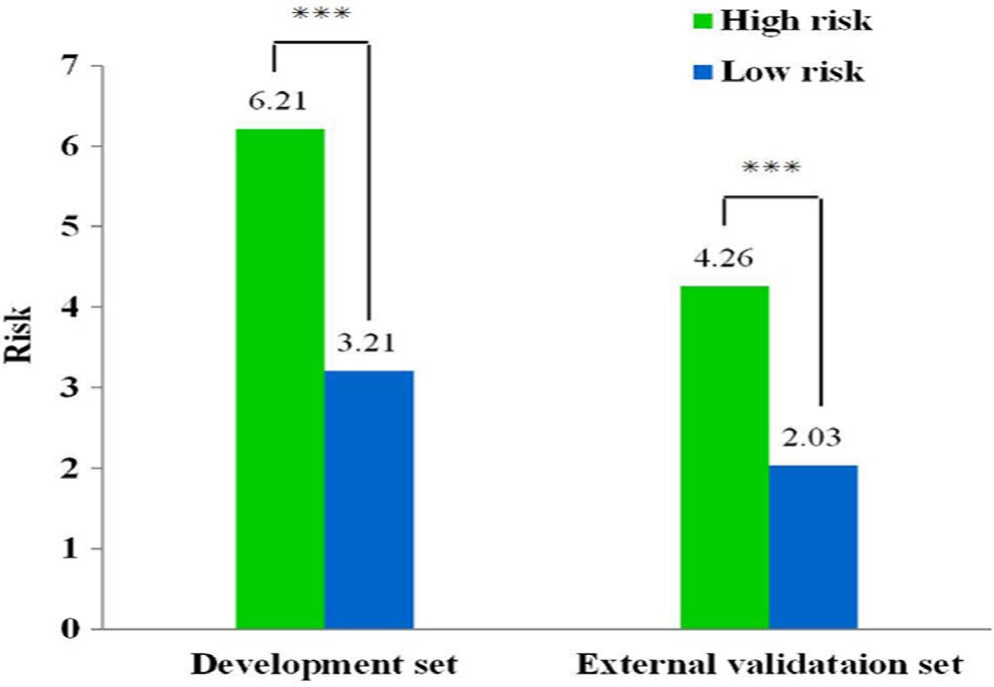
Risk group stratification for detection rate of upper gastrointestinal precancerous lesions

A cutoff value for the UGPL training set were determined by ROC curve, after calculating their total scores (score: 0‐127 as low risk and >127 as high risk for UGPL). Each subgroup (high risk and low risk) showed a distinct detection rate both in development and external validation groups (both *P* < .001; Figure 4).

## 4 DISCUSSION

To our knowledge, nomogram for predicting the risk of UGPL has not been constructed and external validated in non‐high‐risk areas of China. In our study, we analyze the results of UGC screening conducted in the first 7 year of CanSPUC in Hunan province from October 2012 to October 2019. Totally, we reported the results of 35,621 participants undertaking UGC screening among a high‐risk urban population. The analyses showed that the overall participation rate in screening endoscopy among high‐risk population was relatively low (29.1%). For 10,364 participants undertaking endoscopy, the detection rate for UGPL or over, GCPL or over and ECPL or over were 4.55%, 3.79%, and 1.03%, respectively. A nomogram for predicting the risk of UGPL was developed and external validated. The UGPL nomogram involved factors are age, gender, picked food, atrophic gastritis history, and mental trama. The calibration data for UGPL showed optimal agreement between the nomogram prediction and actual observation. The C‐index value of internal and external validation of the nomogram model is 0.612 and 0.670, respectively. Furthermore, high‐risk and low‐risk group divided based on score from the nomogram predicted a significantly distinct detection rate.

The overall participation rate in screening endoscopy among high‐risk population was 29.1%, which is lower than our previous cluster randomized trial (45.6%). In scientific research, the participation rate may be easily overestimated because in the process of implementation community doctors probably could involuntarily select those participants who were more willing to comply with the study protocol. Moreover, to make the study completed almost perfectly, researchers and all staff involved also would put more effort to improve the participant rate through some special methods, like increasing communication with high‐risk participants, taking incentives, even contacting their children to persuade them to accept endoscopy, et al So the 29.1% of participation rate in cross‐sectional study over severe years are more authentic and natural. The rate in our study was lower than the participation rate (53.1%) in high‐incidence areas (Linxian in Henan province, Feicheng in Shandong province, and Cixian in Hebei province).^21^ Since 2005, the Chinese government has implemented a series of screening and early detection programs in 11 high‐risk areas. Public education campaign on the benefit of endoscopic screening has been ongoing for more than ten years in the high‐risk areas while for only several years in the non‐high‐risk areas. This may explain higher participation of endoscopy as part of a general physical examination in high‐risk area. But our rate was higher than the participation rate in another non‐high‐risk area (18.4%) from a recent study in China.^22^ Overall, the compliance of endoscopy in general population is not optimal and is still a major challenge in UGC screening.

As our previous study manifested that PL is the main detection result in non‐high‐risk area as the low detection rate of UGC. So in present study, we reported the PL results and analyze their independent associated risk factor by multivariate logistic analysis. The detection rate of GCPL was 3.79% in our study, which was higher than that of some screening program in China, but lower than that of some studied performed in high‐risk areas. Guo et al reported that the detection rate is 0.41% also in CanSPUC program as non‐high‐risk area by analyzing 7,966 subjects taking endoscopy.^22^ Meanwhile, another study on 12,454 participates with endoscopy presented the detection rate was 0.67%.^23^ However, Zhang et al found that the detection rate of GCPL or over was 4.54% in Henan rural high‐risk area,^24^ and a study conducted in Korea showed the rate was 4.2%.^25^ As for ECPL, our detection rate was lower than several studies. Meng et al reported that the detection rate of esophageal lesions was about 31% in high‐risk area on multi‐center study (Linxian, Feicheng and Cixian).^21^ And in Guo et al study, he found the ECPL detection rate was 3.54%. Therefore, our detection rate of GCPL was relatively high. In future, the area of Hunan province needs to be paid more attention to prevent GCPL which could be intervened effectively by early treatment, thus to reduce the incidence and mortality of GC in the long term.

In multivariate logistic analysis, we found several factors were associated with the risk of PL. Age, and male gender have been confirmed as risk factors for UGC in many severity studies in high‐incidence areas.^26^, ^27^ Picked food and salty food intake frequently were positively correlated with PL, while vegetable intake frequently was associated with a lower risk of GCPL, and evidence of this relationship has been provided in some risk factors studies.^27^, ^28^, ^29^, ^30^ Mental trauma history of participates containing major accidental bodily injury, seriously ill or dies of close relatives, threats of violence et al, may cause easily some physical problems, which push them to see doctors or take physical examination, thus they accepting early treatment or interventions for bad living habits to prevent GCPL and acting as a protective factor. Participations with atrophic gastritis history had higher risk of UCPL. As far as we know, atrophic gastritis is one of the middle stage from normal gastric mucosa progression to hyperplasia and GC. So if the participant with atrophic gastritis who did not accept treatment timely would probably progress to UGPL, even to UGC. Overall, few studies have investigated the risk factors for UGC in non‐high‐incidence areas in China, and the risk factors might be mostly consistent with those of high‐incidence areas. However, other potential risk factors could be considered in future research in urban cities, including tea consumption, vitamin C supplementation, food preservation, salt intake, and biomass smoke exposure.^31^, ^32^, ^33^

Our nomogram showed that age group accounted for the largest contribution to the risk of UGPL. Then, the contributions of the male gender, mental trauma history, picked food intake frequently, and atrophic gastritis history in the nomogram, though significant, were less than that of age group. We found that the C‐index value of our nomogram by external validation was significantly higher than that by internal validation (0.670 vs 0.612). Moreover, the calibration curve showed optimum agreement between the nomogram predictions and actual observations. Those indicated that our nomogram was satisfactory for detecting UGPL from high‐risk participants and had great generalizability as well as reliability for external application. Later, the distinct detection rate of two subgroups validated the distinguishing capacity by cutting total score from the nomogram plot. But the accuracy of the model was not good with the C‐index value less than 0.70. It is acceptable and reasonable for us as the disease could be predicted with the 60% accuracy just in accordance to some oral risk factor questionnaire. And it should be kept in mind though that this study aimed to develop a prescreening tool not a diagnostic tool. Also, this indicated us adding another step evaluation by some another tests, such as serology test, could be required after questionnaire evaluation. In a recent multicentre cross‐sectional study, a predicted model of GC was developed and validated involving serology biomarkers (pepsinogen I, pepsinogen II, anti‐*Helicobacter pylori* antibody, and gastrin‐17) with the C‐index value of 0.76.^29^ But based on hospital opportunistic screening, it would be limited for applying in population screening. Therefore, our next research goal is taking these serology biomarkers applied in our predicted model for targeting the high‐risk UGC population to improve the model accuracy.

Attention needs to be paid to the strengths and limitations of the study when interpreting the results. A major strength is that our study is the first to develop and external validate the prediction model of UGPL on the strength of population‐based endoscopy screening in non‐high‐risk areas with a large‐scale sample. Second, potential risk factors of UGPL analyzed were comprehensive for modeling the nomogram involving the general information, diets intake habit, living habit, psychological state, disease history, and family cancer history in a questionnaire. Moreover, we found a new related factor of metal trauma history with lower risk for UGPL. Furthermore, the data quality was reliable and ensured in two aspects: one hand was our analysis data from CanSPUC supported and monitored by an expert panel of National Cancer Center; on the other hand, data accuracy benefited from information system from the implement process of risk assessment to clinical screening data. However, there are also some limitations. First, the model constructed in high‐risk population might weaken some associations between risk factors involved in the risk assessment and UGPL, such as family history. But it is essential for us to take this analysis for validating preincluded factors and then exploring more accurate risk factors, thus optimizing risk assessment tools for future research. Second, our study was performed just in two cities rather than multiple centers or entire population in Hunan province, so selection bias cannot be ruled out. Third, the C‐index value is relatively low for predicting the risk of UGPL as some serology text variables were not incorporated.

In conclusion, the predicted nomogram developed and external validated in our study has good performance in identifying individuals at a higher risk of UGPL as primary screening before endoscopy among Chinese population in non‐high‐risk areas. And it could greatly improve the screening efficiency by targeting accurately high‐risk population for UGPL. Therefore, it provides us a simple and accurate tool for screening workers to find those who are more likely to be diagnosed as UGPL quickly, thus reducing the incidence and mortality of UGC by improving the detection rate of UCPL and early treatment rate. A future population‐based screening project including serology tests should be carried out to improve this predicting model.

## CONFLICT OF INTEREST

The authors declare no competing interests.

## AUTHOR CONTRIBUTIONS

Haifan Xiao and Xian‐Zhen Liao designed the study. Haifan Xiao, Xian‐Zhen Liao, Shi‐Peng Yan, Ji‐Gang Li, Zhao‐Hui Shi, Yan‐Hua Zou, Ke‐Kui Xu collected and analyzed data. Haifan Xiao wrote the paper. Xian‐Zhen Liao revised the paper. All authors confirmed that the content has not been published elsewhere and does not overlap with or duplicate their published work.

## Supporting information

Fig S1

Table S1

## Data Availability

The data that support the findings of this study are available from Cancer Hospital Chinese Academy of Medical Sciences. Restrictions apply to the availability of these data, which were used under license for this study. Data are available with the permission of Cancer Hospital Chinese Academy of Medical Sciences.
