# Supplementary figures and images for: Development and external validation of a nomogram to predict the risk of Upper gastrointestinal precancerous lesions in a non‐high‐incidence area
Source: Cancer Med. 2020 Sep 16;9(22):8722–32. doi: 10.1002/cam4.3462 (PMC7666758; doi:10.1002/cam4.3462)

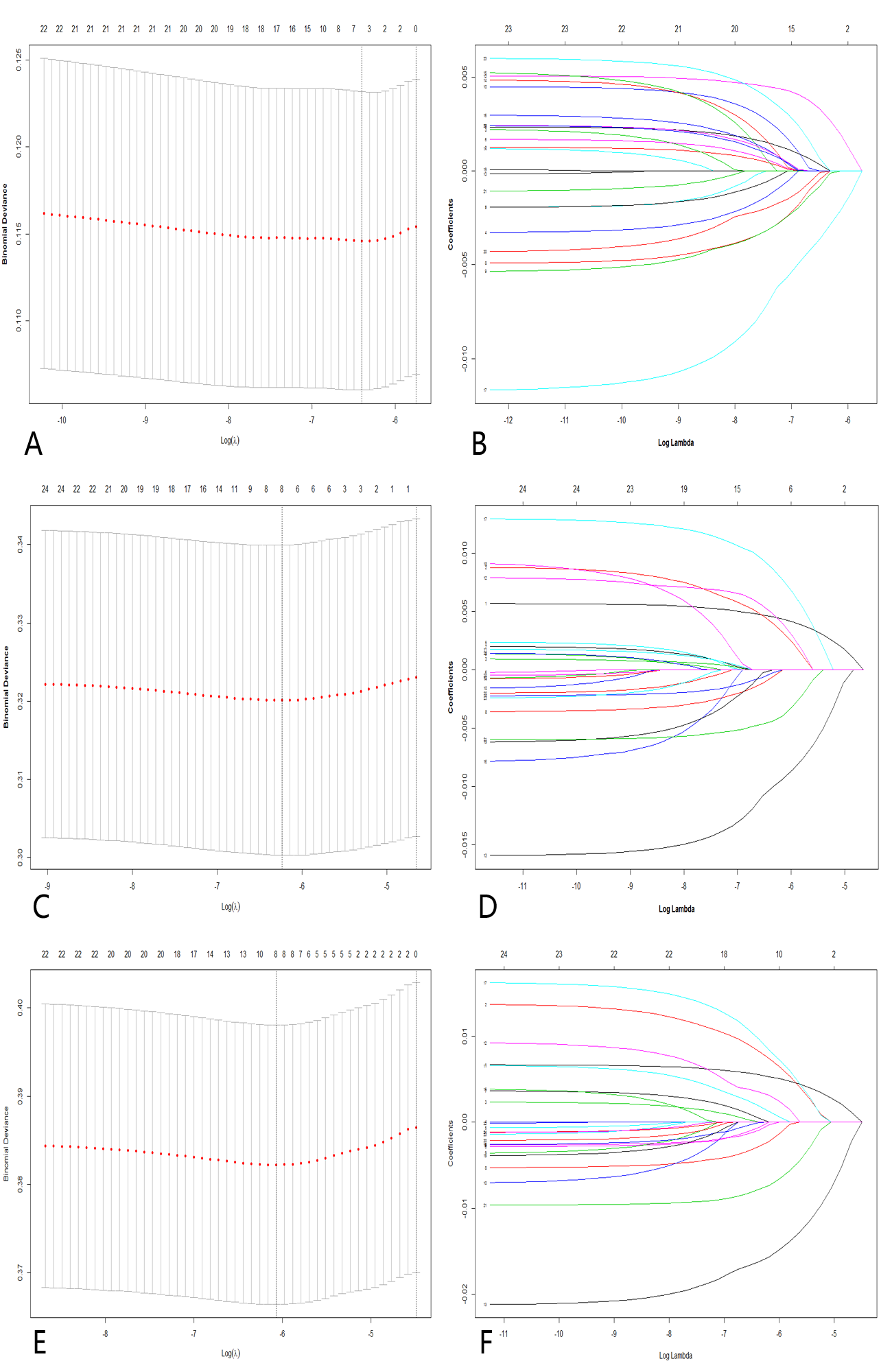

Supplement: Supplementary file 1 — Fig S1 [file CAM4-9-8722-s001.tif]
