# Supplementary material for: Development and external validation of a nomogram to predict the risk of Upper gastrointestinal precancerous lesions in a non‐high‐incidence area
Source: Cancer Med. 2020 Sep 16;9(22):8722–32. doi: 10.1002/cam4.3462 (PMC7666758; doi:10.1002/cam4.3462)
Supplement: Supplementary file 2 — Table S1 [file CAM4-9-8722-s002.docx]

Supplement Table 1 Demographic characteristic variables and potential risk factors for gastric and esophageal precancerous lesions

| Variables |  | GCPL | | |  | ECPL | | |
| --- | --- | --- | --- | --- | --- | --- | --- | --- |
|  |  | Traing set (n=8891) | | Validation set (n=1473) |  | Traing set (n=8891) | | Validation set (n=1473) |
|  |  | Case, % | P | Case, % |  | Case,% | P | Case,% |
| All cases |  | 337 (3.79) |  | 35 (2.38) |  | 92 (1.03) |  | 9 (0.61) |
| Age ^*^ | ‾x ±s | 56.13 ±8.09 |  | 53.37 ±8.24 |  | 56.13 ±8.09 |  | 53.37 ±8.24 |
| Age group (Year) | 40-44 /40-49 | 16 (1.92) | 0.000 | 4 (1.57) |  | 18(0.86) | 0.122 | 0 |
|  | 45-49 /50-59 | 33 (2.62) |  | 4 (1.31) |  | 29(1.75) |  | 3 (0.61) |
|  | 50-54 />59 | 60 (3.41) |  | 7 (2.41) |  | 45(1.31) |  | 6 (1.44) |
|  | 55-59 | 62 (3.85) |  | 8 (3.90) |  | — |  |  |
|  | 60-64 | 87 (4.78) |  | 5 (2.03) |  | — |  |  |
|  | >64 | 79 (4.92) |  | 7 (4.07) |  | — |  |  |
| Body mass index (Kg/m^2^ ) | <18.5 | 19 (6.96) | 0.009 | 0 |  | 2 (0.73) | 0.801 | 0 |
|  | 18.5-23.9 | 183 (3.40) |  | 23 (2.54) |  | 53 (0.98) |  | 6 (0.66) |
|  | 24-26.9 | 100 (4.07) |  | 9 (2.42) |  | 27 (1.10) |  | 2 (0.54) |
|  | ≥27 | 35 (4.54) |  | 3 (2.01) |  | 10 (1.30) |  | 1 (0.68) |
| Gender | Male | 173 (4.65) | 0.000 | 21 (3.38) |  | 52 (1.40) | 0.006 | 6 (0.96) |
|  | Female | 164 (3.17) |  | 14 (1.65) |  | 40 (0.77) |  | 3 (0.35) |
| Marital status | Married | 324 (3.80) | 0.999 | 2 (4.17) |  | 90 (1.05) | 0.589 | 9 (0.63) |
|  | Unmarried | 13 (3.66) |  | 33 (2.32) |  | 2 (0.56) |  | 0 |
| Education | Primary school or below | 47 (3.30) | 0.383 | 5 (1.71) |  | 16 (1.11) | 0.139 | 2 (0.68) |
|  | Middle school | 238 (3.92) |  | 26 (2.78) |  | 54 (0.90) |  | 5 (0.53) |
|  | College degree or above | 52 (3.49) |  | 4 (1.63) |  | 22 (1.48) |  | 2 (0.82) |
| Occupational exposure | Yes | 96 (3.50) | 0.372 | 4 (2.92) |  | 24 (0.88) | 0.380 | 8 (0.60) |
|  | No | 241 (3.92) |  | 31 (2.32) |  | 68 (1.11) |  | 1 (0.73) |
| Smoking | Current | 125 (4.29) | 0.190 | 12 (4.72) |  | 37 (1.08) | 0.116 | 3 (1.18) |
|  | Never | 200 (3.58) |  | 23 (1.89) |  | 54 (1.05) |  | 6 (0.49) |
|  | Former | 12 (3.03) |  | 0 |  | 1 (0.33) |  | 0 |
| Drinking | Current | 132 (3.85) | 0.319 | 8 (4.10) |  | 35 (1.20) | 0.578 | 1 (0.51) |
|  | Never | 189 (3.66) |  | 27 (2.11) |  | 50 (0.90) |  | 8 (0.63) |
|  | Former | 16 (5.35) |  | 0 |  | 7 (1.77) |  | 0 |
| Vegetable intake | Frequently | 224 (3.55) | 0.078 | 32 (2.23) |  | 66 (1.05) | 0.949 | 9 (0.63) |
|  | Rarely | 113 (4.37) |  | 3 (7.69) |  | 26 (1.00) |  | 0 |
| Fruit intake | Frequently | 76 (4.15) | 0.408 | 24 (2.38) |  | 13 (0.71) | 0.157 | 7 (0.69) |
|  | Rarely | 261 (3.70) |  | 11 (2.38) |  | 79 (1.12) |  | 2 (0.43) |
| Meat intake | Frequently | 149 (4.05) | 0.307 | 32 (2.57) |  | 42 (1.14) | 0.465 | 7 (0.56) |
|  | Rarely | 188 (3.61) |  | 3 (1.32) |  | 50 (0.96) |  | 2 (0.88) |
| Coarse Food Grain intake | Frequently | 72 (3.68) | 0.277 | 8 (1.89) |  | 12 (0.71) | 0.188 | 5 (1.18) |
|  | Rarely | 265 (4.28) |  | 27 (2.57) |  | 80 (1.11) |  | 4 (0.38) |
| Pickled food intake | Frequently | 117 (4.65) | 0.009 | 12 (3.85) |  | 27 (1.07) | 0.918 | 3 (0.96) |
|  | Rarely | 220 (3.45) |  | 23 (1.98) |  | 65 (1.02) |  | 6 (0.52) |
| High-temp food intake | Frequently | 131 (3.87) | 0.792 | 3 (1.42) |  | 28 (0.83) | 0.161 | 2 (0.94) |
|  | Rarely | 206 (3.74) |  | 32 (2.54) |  | 64 (1.16) |  | 7 (0.56) |
| Salty food intake | Frequently | 166 (3.59) | 0.326 | 18 (2.87) |  | 62 (1.34) | 0.004 | 3 (0.48) |
|  | Rarely | 171 (4.01) |  | 17 (2.01) |  | 30 (0.70) |  | 6 (0.71) |
| Dry food intake | Frequently | 127 (3.89) | 0.745 | 1 (0.88) |  | 25 (0.77) | 0.072 | 1 (0.88) |
|  | Rarely | 210 (3.73) |  | 34 (2.50) |  | 67 (1.19) |  | 8 (0.59) |
| Fried food intake | Frequently | 163 (3.79) | 0.999 | 0 |  | 40 (0.93) | 0.403 | 2 (1.57) |
|  | Rarely | 174 (3.79) |  | 35 (2.60) |  | 52 (1.13) |  | 7 (0.52) |
| Mind breakdown recently | Yes | 87 (3.16) | 0.042 | 1 (1.61) |  | 21 (0.76) | 0.112 | 0 |
|  | No | 250 (4.07) |  | 34 (2.41) |  | 71 (1.16) |  | 9 (0.64) |
| Chronic mental depression | Yes | 92 (3.26) | 0.087 | 3 (4.69) |  | 26 (0.92) | 0.548 | 0 |
|  | No | 245 (4.03) |  | 32 (2.27) |  | 66 (1.09) |  | 9 (0.64) |
| Reflux esophagitis | Yes | 103 (3.92) | 0.722 | 2 (4.35) |  | 30 (1.14) | 0.595 | 1 (0.22) |
|  | No | 234 (3.74) |  | 33 (2.31) |  | 62 (0.99) |  | 8 (0.56) |
| Superficial gastritis | Yes | 256 (3.73) | 0.649 | 7 (1.70) |  | 70 (1.02) | 0.908 | 4 (0.97) |
|  | No | 81 (3.98) |  | 28 (2.64) |  | 22(1.08) |  | 5 (0.47) |
| Atrophic gastritis | Yes | 137 (4.16) | 0.075 | 1 (4.76) |  | 41 (1.28) | 0.105 | 0 |
|  | No | 200 (3.51) |  | 34 (2.34) |  | 51 (0.90) |  | 9 (0.62) |
| Gastric and duodenal ulcers | Yes | 231 (3.88) | 0.572 | 8 (3.39) |  | 60 (1.01) | 0.803 | 1 (0.42) |
|  | No | 106 (3.61) |  | 27 (2.18) |  | 32 (1.09) |  | 8 (0.65) |
| Family history of gastric cancer | Yes | 222 (3.78) | 0.969 | 2 (3.57) |  | — |  | — |
|  | No | 115 (3.82) |  | 33 (2.32) |  | — |  | — |
| Family history of esophageal cancer | Yes | — |  | — |  | 12 (1.58) | 0.172 | 0 |
|  | No | — |  | — |  | 80 (0.98) |  | 9 (0.62) |

* Variabls were described by mean (**x**) and standard deviation(**s**).

**Supplement Figure Legends**

**Figure 1.** Demographic and potential risk factors identification and selection by LASSO regression model. Ten-fold cross–validation was applied to select the most suitable parameter using LASSO regression model for UGPL (A), GCPL (C) and ECPL (E). Coefficient curves of the 24 parameters for UGPL(B) and GCPL (D), and 23 parameters for ECPL (F).

LASSO, the least absolute shrinkage and selection operator; UGPL, upper gastrointestinal precancerous lesions; GCPL, gastric cancer precancerous lesions; ECPL, esophageal cancer precancerous lesions
